# Mycobacterial Prevalence and Antibiotic Resistance Frequency Trends in Taiwan of Mycobacterial Clinical Isolates From 2002 to 2014

**DOI:** 10.1097/MD.0000000000002942

**Published:** 2016-03-25

**Authors:** Ming-Yuh Shiau, Ming-Shih Lee, Tian-Lin Huang, Jen-Ning Tsai, Yih-Hsin Chang

**Affiliations:** From the Department of Nursing (M-YS), College of Medicine and Nursing, Hungkuang University, Taichung; Clinical Laboratory (M-SL, T-LH), Chung Shan Medical University-Hospital, Taichung; School of Medical Laboratory and Biotechnology (M-SL, J-NT), Chung Shan Medical University, Taichung; and Department of Biotechnology and Laboratory Science in Medicine (Y-HC), National Yang-Ming University, Taipei, Taiwan.

## Abstract

Tuberculosis, caused by *Mycobacterium tuberculosis* complex (MTBC) infections, is one of the most widespread infectious diseases worldwide. Nontuberculous mycobacteria (NTM) also cause chronic pulmonary infections, however, NTM infection is generally overlooked.

This study analyzed the frequencies of MTBC and NTM clinical isolates from 181,132 specimens obtained from patients in Taiwan suspected of having a pulmonary mycobacterial infection from 2002 to 2014. The resistant rates to 4 first-line antibiotics (isoniazid, ethambutol, rifampicin, and streptomycin) of 9079 clinical MTBC isolates were also examined by the modified agar proportion method.

Overall, the mycobacterial isolation rate was 8.65%, and this consisted of MTBC isolation rate of 5.01% and NTM isolation rate of 3.63%. The prevalence of MTBC isolates among the identified mycobacterial strains could be seen to decrease significantly from 82.5% in 2002 to 41.18% in 2014. Notably, the corresponding NTM prevalence increased 3.36 fold from 17.54% in 2002 to 58.82% in 2014. The frequencies of MTBC and NTM isolates showed a reciprocal trend with the crossing over occurring in the years 2010 and 2011. Although the resistance rates of the MTBC isolates to isoniazid and streptomycin were relatively stable over the study period, resistance rates of the MTBC isolates against rifampicin and ethambutol fluctuated across the study period. Overall, the incidence of multidrug resistance was relatively consistent at about 1.74%.

The diagnosis, identification, and susceptibility tests for NTM should be standardized and integrated into appropriate clinical settings to cope with the increase in NTM infections. In addition, the documentation of the antibiotic resistance rates of MTBC clinical isolates to the antibiotic treatments most often clinically prescribed over a decade provides valuable clues and reference points for effective mycobacterial control.

## INTRODUCTION

Tuberculosis (TB), caused by *Mycobacterium tuberculosis* complex (MTBC) infection, is one of the most widespread infectious diseases and remains one of the leading public health problems worldwide.^1^ The emergence of drug resistance has long been a major hindrance in relation to TB control, especially multidrug resistance (MDR). According to World Health Organization statistics, about 2 billion persons have suffered from TB infection globally and the estimated incidence of TB was approximately 9 million cases in 2013, including 480,000 cases with MDR TB and 1.5 million TB deaths.^1^ In addition to being difficult to treat, the treatment outcome when patients have MDR TB is very likely to be failure and death.

TB is also one of the most dangerous communicable diseases in Taiwan with the highest incidence and mortality rate; despite the execution of a national TB control program since 2006.^2^ In 2012, 12,338 TB cases including 1.1% MDR TB cases and 626 TB-related deaths were reported. Environmental hygiene conditions, population density, health care resources, and so on, are all major factors dictating TB incidence, mortality, and drug resistance rates across different geographical areas.

Nontuberculous mycobacteria (NTM) are opportunistic pathogens that cause skin and chronic pulmonary infection. Owing to the similar clinical pulmonary syndrome and x-ray manifestations of NTM infection to MTBC, NTM does not receive as much attention as TB. As a matter of fact, NTM infection is generally overlooked. Our previous study documented the identification of 5349 MTBC and 2675 NTM clinical isolates from 99,200 specimens of patients suspected of having a mycobacterial infection from 2002 to 2007.^3^ Notably, a reciprocal trend of MTBC and NTM prevalence was identified, with the NTM isolation rate increasing 2.6 fold during the study period. We suggested that the diagnosis, identification, and susceptibility tests for NTM should be standardized and integrated as the standard operation protocols in clinical settings and laboratories to cope with the increase in NTM infections.

Taking in to account the above context, the present study has analyzed and reports the frequencies of MTBC and NTM clinical isolates from 2002 to 2014 to further monitor mycobacterial infection trends. Notwithstanding the fact that the frequency of isolation of MTBC showed a declining trend in our previous study,^3^ information regarding MTBC drug resistance remained pivotal and provides valuable findings that help the effective prescription of appropriate treatments and help the implementation of optimal strategies to control TB infection. Accordingly, the resistant rates to the 4 first-line antibiotics, namely isoniazid (INH), rifampicin (RMP), ethambutol (EMB), and streptomycin (SM), of the clinical MTBC isolates during the study period are also reported. This study documents mycobacterial infection and drug resistance data that have accumulated for more than a decade and, therefore, are able to provide important reference points for implementing optimal intervention strategies for the control of mycobacterial infection.

## METHODS

### Sample Collection and Mycobacterial Isolation

The clinical isolates in the present study were collected and identified from year 2002 to 2014. The population from which the samples were collected resides in the central Taiwan area including Taichung City, Miaoli County, Nantou County, and Changhua County; the population of this area is >5.1 million, which corresponds to 21.74% of the total population living in Taiwan. Samples from patients suspected to be infected with *Mycobacterium* and who had been admitted to a hospital or clinic (including medical centers, regional hospitals, clinics, medical laboratories, and health centers) in the above areas were collected, then submitted to the TB center of Chung Shan Medical University Hospital for further processing, bacterial culture, identification, and antibiotic susceptibility testing. A total of 181,132 specimens, including 165,590 sputum samples (91.42%), 3772 pleural effusion samples (2.08%), 2887 bronchial-alveolar lavage samples (BAL, 1.59%) and various other samples were collected (these are listed in supplementary Table 1). The procedures used on all samples collected conformed to the standard protocol and guidelines from Department of Health, Taiwan.^3,4^ Ethical approval was not required because of the basis of the retrospective nature of this study.

### Antibiotic Susceptibility Testing Modified Agar Proportion Method

Quadrant Middlebrook 7H10 (for standard testing) or 7H11 (for INH-R stains) agar plates supplemented with 10% oleic acid-albumin-dextrose-catalase were used to test the antibiotic resistance of the isolated strains using *M tuberculosis* H37Rv (ATCC27294) as the QC strain. Resistance was defined as the growth on drug-containing quadrants that was >1% of an inoculum of bacterial cells in the presence of a “critical concentration” of agent (inhibits 95% of MTBC wild strains while not inhibiting MTBC strains from patients who have failed therapy).^5^

## RESULTS AND DISCUSSION

To allow further monitoring and to address the trend in mycobacterial infection, the clinical isolation rates of MTBC and NTM during 2002 to 2014 were investigated. In addition, profiling of the resistance rates of MTBC clinical isolates to 4 first-line antibiotics, namely INH (0.2 and 1 μg/mL, INH-0.2, and INH-1), RMP (1 μg/mL, RMP-1), EMB (5 μg/mL, EMB-5), and SM (2 and 10 μg/mL, SM-2, and SM-10), were also carried to document MTBC antibiotic resistance over the study period.

### Positive Isolation Rates With Respect to Mycobacterial Cultures

A total of 181,132 specimens were collected over the 13-year study period. Among these specimens, 15,662 mycobacterial isolates were obtained, including 9079 MTBC and 6583 NTM. The numbers of clinical specimens that were positive for MTBC and NTM isolation and the corresponding positive isolation rates are respectively listed in Table 1 and Table 2. The overall mycobacterial isolation rate was 8.65% (15,662/181,132), with the overall MTBC and NTM isolation rates being 5.01% (9079/181,132) and 3.63% (6583/181,132), respectively. The MTBC isolation rates ranged from 3.16% (399/12,633, year 2010) and 6.20% (1140/18,385, year 2005) over the study period. Notably, the NTM positive isolation rates increased year by year from 1.28% (147/11,414, year 2002) to 7.46% (647/8669, year 2014).

**TABLE 1:**
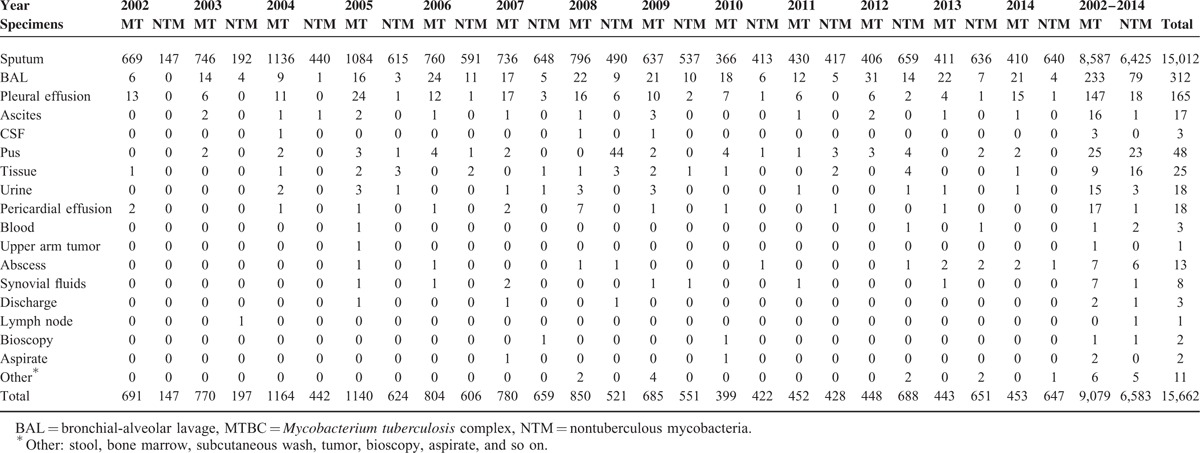
Categories and Numbers of Specimens With Positive MTBC and NTM Isolation in Year 2002 to 2014

**TABLE 2:**
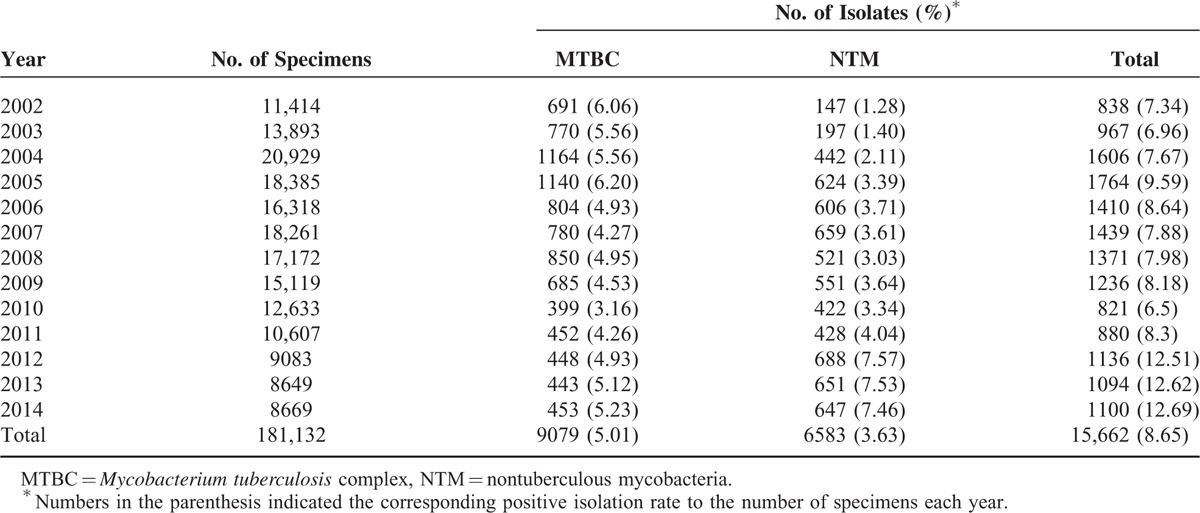
Positive Isolation Rate of Mycobacterial Culture From Year 2002 to 2014

### Distribution of MTB and NTM Isolates

**FIGURE 1:**
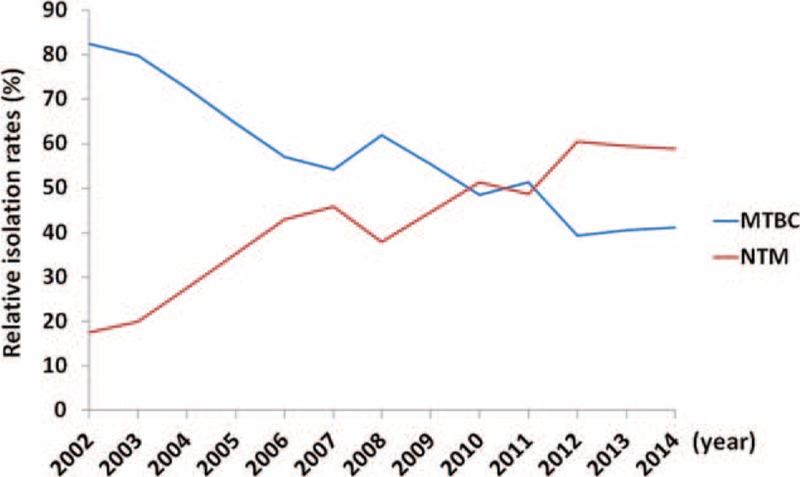
Reciprocal alteration in the relative isolation rates (% in y-axis) of MTBC strains and NTM strains from 2002 to 2014 (x-axis), with the cross-over in the years 2010 and 2011. MTBC = *Mycobacterium tuberculosis* complex, NTM = nontuberculous mycobacteria.

The distribution of MTBC and NTM among our clinical mycobacterial isolates was analyzed and is depicted in Figure 1. Among the 15,662 clinical mycobacterial isolates, the frequency of MTBC isolates showed a consistent decreasing trend as the ratios decreased from 82.50% (691/838) in 2002 to 41.18% (453/1,100) in 2014. In contrast, there was a remarkable trend toward a growth in NTM isolates, with the ratio increasing 3.36 fold from 17.50% (147/838) in 2002 to 58.82% (647/1100) in 2014 being observed. Particularly, the frequencies of MTBC and NTM isolates during these 13 years showed a reciprocal change that crossed over in years 2010 to 2011.

Our results reveal a significant reciprocal alteration in the MTBC and NTM isolation rates in central Taiwan during the study period with there being a concurrent decline in the incidence of MTBC isolation and parallel increase in the incidence of NTM isolation. These observations support our previous findings as well as conclusions from other reports, namely that an increase in the isolation of NTM clinical specimens is accompanied by a decrease in the incidence of TB.^6–10^ The possible factors leading to this trend toward a growth in the isolation of NTM strains include an increased exposure of predisposed individuals, increase recognition of NTM infection by physicians and microbiologists, the implementation of public health strategies and network targeting TB, and improvements in the diagnostic techniques used in medical laboratory. Moreover, the current findings further support our previous suggestion that the availability of new techniques that allow accurate identification of NTM strains may result in a further increase of NTM diagnosis in the future.^3^

The clinical manifestations of MTBC and NTM infections frequently overlap, which is the major difficulty that must be overcome when specifically diagnosing NTM infections.^11,12^ In addition, the consistent increase in NTM infections challenges the implement of the directly observed treatment, short course (DOTS) program, a strategy whereby TB is treated according to the positive results from sputum smear acid-fast bacilli microscopy test. As a result of the DOTS program, most NTM-infected patients are very likely to be treated with the most prescribed antituberculous agents when acid-fast bacilli have been detected in their sputum smear before the confirmative NTM identification results are available. Unfortunately, this may lead to treatment failure or disease relapse since differences regarding bacterial components, drug-resistance patterns, clinical manifestations, and treatment outcome between NTM and MTBC infection are indeed existed despite of some similarities.^13–15^ Therefore, once NTM is no longer detected in the sputum from patients with a suspected infection, successful treatment and a good outcome may be falsely determined.^16,17^ The abovementioned difficulties that range from the initial diagnosis to the treatment outcome are all possible factors that might lead to the increase and spread of NTM strains, which seems to be reflected in the climbing prevalence identified in the present study.

In this context, the early and precise diagnosis of patients with NTM infection is the prerequisite to prescribe an effective intervention. A standard operation protocol concerning the diagnosis, identification, and susceptibility testing for NTM needs to be established and included in medical laboratories to improve the early diagnosis of NTM infection; this becomes more urgent because the frequency of NTM isolates is consistently and significantly increasing. In addition, the possible underlying factors for this increase must be investigated and uncovered to allow the implementation of public health strategies that are able to effectively control these emerging NTM infections.

### Antibiotic Resistance Profiling of the MTBC Clinical Isolates

**FIGURE 2:**
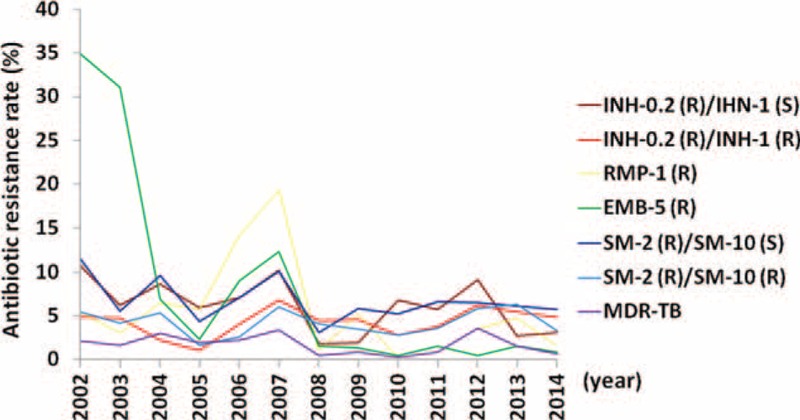
Resistance rates (% in y-axis) of 9079 MTBC clinical isolates against the 4 first-line antituberculous drugs, namely INH, RMP, EMB, and SM during study years (x-axis). EMB = ethambutol, MTBC = *Mycobacterium tuberculosis* complex, INH = isoniazid, RMP = rifampicin, SM = streptomycin.

Among the 9079 MTBC isolates, the INH-0.2 and INH-1 resistant rates were relatively stable averaging 6.15% and 4.30%, respectively (Table 3 and Figure 2). A similar trend was observed for SM-2 and SM-10 resistance, which had average rates of 6.71% and 4.20%, respectively. This contrasts with the dramatic changes detected in RMP and EMB resistance rates that were observed, with the RMP resistance rate ranging from 0.25% in 2010 to 19.34% in 2007 (4.89% on average) and the EMB resistance rate significantly decreasing from 34.9% in 2002 to 0.88% in 2014 (8.04% on average). Finally, the incidence of MDR TB was consistent during the study period with the average rate being 1.74%.

**TABLE 3:**
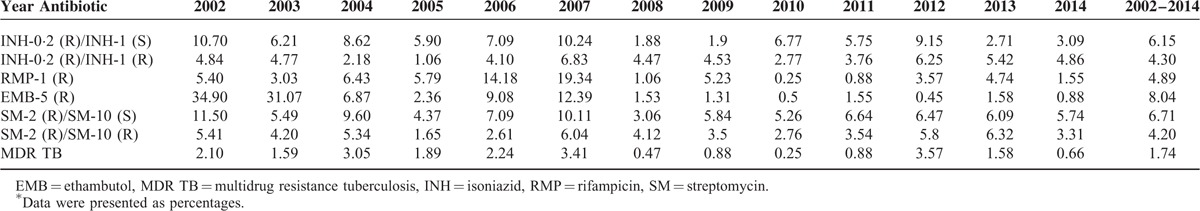
Resistance Rate of MTBC Clinical Isolates Against First-Line Antituberculosis Drugs From Year 2002 to 2014

When these MTBC isolates were stratified by their resistance to the number of first-line drugs they were resistant to (Table 4), approximately 76.03% of the isolates were fully susceptible to the 4 drugs tested. Except for 2003, the frequencies of MTBC isolates that showed resistance to 1, 2, 3, 4, or any 1 of the 4 drugs remained consistent with average rates of 14.94%, 3.80%, 1.01%, 0.56%, and 20.3%, respectively. It should be noted, however, that the resistance rates to a single antibiotic and to any 1 of the 4 drugs in 2003 and 2006 were exceptionally high (31.95% and 42.6% in 2003 and 17.41% and 26.00% in 2006, respectively).

**TABLE 4:**
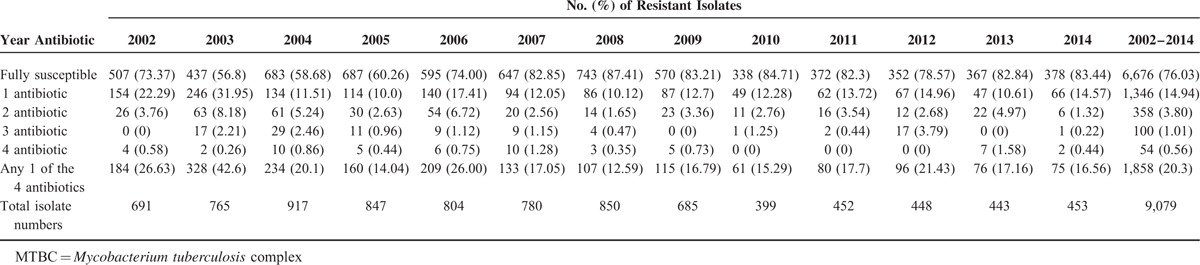
Isolate numbers and percentages of MTBC clinical isolates with resistance to the 4 first-line antituberculosis agents

The issue of ongoing MDR TB spread, which makes TB incurable and leads to patient death, is a study focus globally. About 5% of global TB cases have MDR TB with an estimated 3.5% MDR TB incidence among new TB cases.^1^ The median prevalence of resistance to any of the first-line drugs in patients who have never been treated, most commonly SM and/or INH, was 10.7% (range 0%–57.1%); furthermore, the median prevalence of MDR TB was 1.2% (range 0%–14.2%). Notwithstanding the above, the corresponding figures for patients who had previously been treated were much higher at 23.3% (range 0%–82.1%) and 7.7% (range 0%–58.3%), respectively.^18,19^ Although the MDR TB prevalence found in our study is quite consistent at about 1.74% (Table 3), which is lower than the estimated global MDR TB incidence, the average resistant rates to RMP and EMB were unexpectedly high, with average rates 4.89% and 8.04%, respectively.

We suggest that the resistance rates for the years 2003 to 2007 are a unique phenomenon: the >30% EMB resistance rates in year 2002 to 2003 plunged to <10% within 2 years, but increased again during 2006 to 2007. Since then the rate has remained relatively stable. The alterations in the pattern of EMB-resistance parallel the changes in resistant rates to any 1 of the 4 drugs in the corresponding years (Table 4 and Figure 2). Nevertheless, the MDR TB rates did remain stable owing to the limited alterations of INH and SM resistance rates. Nevertheless, the present study is not able to provide data regarding the incidence among the infected population stratified by treatment regimen; this is due to the nature of this study, namely one based on clinical isolates.

Despite global efforts to reduce TB case numbers, the spread of MDR TB is still a threat that leads to the spread of TB and significant numbers of fatalities.^19^ Treatment of MDR TB is an expensive and prolonged task for both patients and physicians, and it is often associated with a high incidence of noncompliance and adverse reactions. In Taiwan, the TB caseload and incidence rates fell 25.1% and 26.8%, respectively, compared with the corresponding figures prior to the implementation of the “Mobilization Plan to Halve Tuberculosis Incidence in Ten Years” in 2006.^2^ This decline has, however, been slowing down recently. Nevertheless, the reported 70.4% treatment success rate of new smear-positive cases in 2011 is still much lower than that of the WHO target treatment success rate of 85%. Therefore, greater effort and more effective strategies are needed regarding TB control in Taiwan.

Further knowledge and information on mycobacterial prevalence, antibiotic resistance trends, advanced identification methods, and drug susceptibility testing are critical to the effective control of mycobacterial infection. According to our findings concerning mycobacterial infection and drug susceptibility that we have documented over 13 years during the present study, we conclude and suggest that diagnosis, identification, and susceptibility testing for NTM should be standardized and integrated into the clinical settings. In addition, our results for MTBC antibiotic resistant rates provide information and guidance regarding the choice of treatment regimen. Although there are limitations to our study because of its clinical isolate-based nature, our findings have revealed that the trend in mycobacterial infection and drug resistance should provide valuable reference points regarding controlling antimycobacterial drug resistance and the mycobacterial epidemic in Taiwan.

## Supplementary Material

Supplemental Digital Content
