# Supplementary material for: Mycobacterial Prevalence and Antibiotic Resistance Frequency Trends in Taiwan of Mycobacterial Clinical Isolates From 2002 to 2014
Source: Medicine (Baltimore). 2016 Mar 25;95(12):e2942. doi: 10.1097/MD.0000000000002942 (PMC4998363; doi:10.1097/MD.0000000000002942)
Supplement: Supplemental Digital Content [file medi-95-e2942-s001.pdf]

Supplemental Table 1. Categories and numbers of specimens for mycobacterial culture in year 2002-2014

Year / Specimens	2002	2003	2004	2005	2006	2007	2008	2009	2010	2011	2012	2013	2014	2002-2014	
														n	(%)
Sputum	10,349	12,759	19,602	17,179	15,068	16,764	15,402	13,629	11,431	9,767	8,217	7,720	7,698	165,590	91·42
Pleural effusion	454	593	758	691	752	865	993	767	564	301	287	409	451	3,772	2·08
BAL	167	180	184	180	176	246	231	278	224	263	240	245	273	2,887	1·59
CSF	98	91	128	79	88	107	123	113	118	69	72	51	30	1,167	0·64
Urine	89	97	63	95	59	58	60	61	72	21	38	13	28	754	0·42
Ascites	38	91	100	79	89	68	103	68	48	41	33	55	18	831	0·46
Pus	44	25	47	41	45	49	77	72	56	40	31	12	24	563	0·31
Endotracheal aspirate	100	20	0	0	0	0	9	0	12	0	7	9	0	157	0·09
Synovial fluid	3	14	19	15	14	27	47	33	36	43	39	40	55	385	0·21
Pericardial effusion	5	16	11	15	8	13	26	15	12	6	5	6	5	143	0·08
Blood	10	5	11	4	12	5	4	6	6	5	4	9	6	87	0·05
Tissue	19	1	5	5	1	12	33	38	24	26	34	29	22	249	0·14
Abscess	2	0	1	0	5	11	4	8	3	11	14	14	23	96	0·05
Discharge	12	0	0	1	0	1	7	1	0	0	2	0	0	24	0·01
Body fluid	7	0	0	0	0	6	11	9	8	6	5	7	23	82	0·04
Gastric juice	2	0	0	0	0	5	16	12	7	3	5	3	3	56	0·03
Throat swab	6	0	0	0	0	0	2	0	0	0	0	0	0	8	<0·01
Other*	9	1	0	1	1	24	24	9	12	5	50	27	10	173	0·09
Total	11,414	13,893	20,929	18,385	16,318	18,261	17,172	15,119	12,633	10,607	9,083	8,649	8,669	181,132	100

*Other: stool, bone marrow, subcutaneous wash, tumor, bioscopy, aspirate, etc.

BAL: bronchial alveolar lavage; CSF: cerebrospinal fluid.
